# Simulation of mass spectrometry-based proteomics data with Synthedia

**DOI:** 10.1093/bioadv/vbac096

**Published:** 2022-12-19

**Authors:** Michael G Leeming, Ching-Seng Ang, Shuai Nie, Swati Varshney, Nicholas A Williamson

**Affiliations:** Bio21 Molecular Science & Biotechnology Institute, Melbourne Mass Spectrometry and Proteomics Facility, The University of Melbourne, Melbourne, VIC 3052, Australia; Bio21 Molecular Science & Biotechnology Institute, Melbourne Mass Spectrometry and Proteomics Facility, The University of Melbourne, Melbourne, VIC 3052, Australia; Bio21 Molecular Science & Biotechnology Institute, Melbourne Mass Spectrometry and Proteomics Facility, The University of Melbourne, Melbourne, VIC 3052, Australia; Bio21 Molecular Science & Biotechnology Institute, Melbourne Mass Spectrometry and Proteomics Facility, The University of Melbourne, Melbourne, VIC 3052, Australia; Bio21 Molecular Science & Biotechnology Institute, Melbourne Mass Spectrometry and Proteomics Facility, The University of Melbourne, Melbourne, VIC 3052, Australia

## Abstract

**Motivation:**

A large number of experimental and bioinformatic parameters must be set to identify and quantify peptides in mass spectrometry experiments and each of these will impact the results. An ability to simulate raw data with known contents would allow researchers to rapidly explore the effects of varying experimental parameters and systematically investigate downstream processing software. A range of data simulators are available for established data-dependent acquisition methodologies, but these do not extend to the rapidly developing field of data-independent acquisition (DIA) strategies.

**Results:**

Here, we present *Synthedia—*a software package to simulate DIA liquid chromatography-mass spectrometry for bottom-up proteomics experiments. Synthedia can generate datasets with known peptide precursor ions and fragments and allows for the customization of a wide variety of chromatographic and mass spectrometry parameters.

**Availability and implementation:**

Synthedia is freely available via the internet and can be used through a graphical website (https://synthedia.org/) or locally via the command line (https://github.com/mgleeming/synthedia/).

**Supplementary information:**

Supplementary data are available at *Bioinformatics Advances* online.

## 1 Introduction

Comprehensive identification and accurate quantitation of peptides is the key goal of mass spectrometry-based proteomics. To achieve as complete data as possible, researchers need to configure a large number of settings and parameters when conducting both experimental analysis of samples as well as bioinformatic processing of the raw data. Importantly though, these instrumental and bioinformatic processing parameters do not operate in isolation and a favourable change in one parameter frequently has a detrimental impact on the experiment elsewhere. For example, increasing the number of fragmentation scan events (that are used to determine the sequence of an observed peptide ion) may provide more opportunities to identify low-abundance peptides; however, quantitative accuracy may suffer since the increased cycle time reduces the number of data points that can be acquired across a chromatographic peak. Optimizing an experimental and bioinformatic analysis pipeline involves finding a balance between parameters that give acceptable results and a substantial literature has been produced in support of this endeavour.

Method optimization procedures are frequently constrained by limited available instrument time, the vast permutation space of possible parameters, and a desire to proceed to the experimental study. To help researchers optimize analytical methods while minimizing time-consuming experimental analyses, computational platforms have been developed that construct liquid chromatography-mass spectrometry/mass spectrometry (LC-MS/MS) data *in silico* (Awan and Saeed, 2018; Bielow *et al.*, 2011; Goldfarb *et al.*, 2016; Hutchins *et al.*, 2019; Kösters *et al.*, 2021; Noyce *et al.*, 2013; Smith and Prince, 2015). These include MSSimulator (Bielow *et al.*, 2011) and SMITER (Kösters *et al.*, 2021) which simulate data-dependent acquisition (DDA) bottom-up proteomics data, Mspire-Simulator (Noyce *et al.*, 2013) which simulates MS1 data with particular attention to noise reproduction and Lipid-DDA-Simulator (Hutchins *et al.*, 2019) which models shotgun MS/MS lipidomics datasets.

These existing data simulators have largely focused on modelling established DDA acquisition methodologies wherein the most abundant peptide ions are selected for fragmentation. In recent years, data-independent acquisition (DIA) methods have gained popularity due to their depth of proteome coverage and robust quantitation (Doerr, 2015). In DIA, a series of pre-defined *m/z* ‘windows’ are sequentially isolated and all precursors ions within a window are simultaneously fragmented regardless of their identity or abundance. MS/MS spectra in DIA methods are therefore highly multiplexed due to the intentional co-isolation and fragmentation of a large number of peptide precursor ions. Substantial post-acquisition computational processing is required to deconvolute multiplexed fragmentation spectra and reveal the identities of detected peptides. Data processing software for DIA proteomics is rapidly evolving and major packages include Spectronaut (Bernhardt *et al.*, 2014), Encyclopedia (Searle *et al.*, 2018), DIA-NN (Demichev *et al.*, 2020) and DIA-Umpire (Tsou *et al.*, 2015).

DIA methods have offered the possibility of deep proteome characterization and quantitation on complex samples even with short chromatographic gradients (<30 min) (Skowronek *et al.*, 2022). However, the interplay between experimental factors (e.g. sample complexity, gradient length and data points per chromatographic peak) and downstream post-acquisition processing outcomes (e.g. peptide identification rates and quantitative accuracy) for DIA is complex and still being actively explored (Gotti *et al.*, 2021; Oliinyk and Meier, 2022). Thus, an ability to generate ground-truth DIA data with a known complement of peptide ions and quantities would be beneficial.

Here, we present ‘Synthedia’ which artificially creates DIA LC-MS/MS data files in ‘.mzML’ format with a defined and customizable peptide complement. A wide range of different operating parameters can be simulated such that detailed investigations can be made as to the expected effects of changing instrument acquisition variables. Moreover, since the identity and abundance of peptides in the simulated data are exactly known, thorough assessment of downstream processing software can be conducted.

## 2 Implementation and usage

Synthedia requires users to specify an input set of peptide precursor ions and MS2 fragmentation products for each precursor to be simulated. These can be given as either the processing output (i.e. the ‘txt’ directory) of a MaxQuant search (Tyanova *et al.*, 2016), or a spectral library predicted by Prosit (Gessulat *et al.*, 2019). To generate simulated peaks, isotope distributions for MS1 are generated for precursor ions given in MaxQuant or Prosit input files using Pyteomics (Goloborodko *et al.*, 2013). Relative retention times for input peptides are determined from experimental values in MaxQuant input data and calculated iRT values in the case of Prosit input data. Chromatographic elution profiles are then modelled as exponentially modified Gaussian distributions and mass spectral peaks can be modelled as peak centroids or, alternatively, as either Lorentzian, Gaussian or exponentially modified Gaussian distributions if full profile data is desired. MS2 fragmentation patterns for each peptide precursor ion are drawn from input MaxQuant or Prosit data. To create MS2 spectra, fragment ions are collected for all precursors within a given DIA isolation window and their intensities are weighted by a factor derived from the chromatographic elution profile of the intact peptide.

Many options can be configured to create mzML files with different characteristics. For example, the same set of peptide precursor ions and fragmentation products can be simulated over different chromatographic gradients, with different chromatographic peak widths and tailing properties. Instability in ionization can be introduced and mass spectral peaks can be simulated with different resolutions, over different scan ranges, at different scan speeds and with different mass accuracies. A full list of configurable parameters and their descriptions is given in Supplementary Table S1.

A wide range of different DIA windowing schemes has been implemented in the acquisition of experimental data (Pino *et al.*, 2020). The default Synthedia simulation creates a series of fixed-width, non-overlapping windows. To create data with custom windowing schemes, users can supply an ‘acquisition schema’ file that explicitly defines each DIA window. By doing so, complex acquisition strategies such as staggered, overlapping and variable-width windows can be simulated. An example acquisition schema file is given in Supplementary Table S2.

To assess quantitative results produced by raw data analysis software, Synthedia can generate datasets containing multiple mzML files that mimic the acquisition of replicate samples from different treatment groups. Here, within and between group variations in individual precursor and fragment abundances are randomly drawn from customizable distributions. Absent or undetectable peptides can also be simulated in a multi-sample comparison by differentially omitting certain ions from some files. These can be configured as group-specific (wherein a peptide is selectively absent in a given group) or sample-specific (where peptides are randomly absent across all samples) manner.

‘Decoy’ precursors and fragments (i.e. detected ions that do not arise from peptides) can optionally be introduced to the simulated mzML files to mimic chemical contaminants and noise. These can be specified by supplying a NIST ‘.msp’ format spectral library containing desired decoy entities. For example, the MS-DIAL team maintains a range of .msp libraries containing lipid, metabolite and xenobiotic spectra (Tsugawa *et al.*, 2015).

The output of a simulation is (i) one or more mzML files, (ii) a text file containing the sequence, retention time, charge, mass-to-charge ratio and abundance values for the ions simulated, (iii) a file that can be used to repeat a given simulation and (iv) a yaml file containing simulation parameters. Synthedia is implemented in Python using the OpenMS framework (Sturm *et al.*, 2008) to manipulate mzML files. The package can be utilized locally or via a web server. The Python package is registered with the Python Package Index and can be easily installed using the ‘pip’ package manager and documentation and usage instructions are available at the GitHub repository. For the web server, a tutorial is included in the Supplementary information. Running time for a simulation depends heavily on the number of processing cores available, the number of precursors and fragments to simulate, and the parameter set. As a guide, a Prosit library with ∼35 000 precursors could be simulated in approximately 1 h for profile and 15 min for centroid using a single processing core on a laptop computer. A detailed breakdown of running times under different conditions is given in Supplementary Table S3. A ‘preview’ function is available allows users to rapidly simulate and visualize a single peptide precursor from the command line that can aid in verifying input parameters prior to running a more time-consuming simulation.

The files produced have been tested with various software that accepts mzML files including DIA-NN, EncyclopeDIA, DIA-Umpire and Skyline (Demichev *et al.*, 2020; MacLean *et al.*, 2010; Searle *et al.*, 2018; Tsou *et al.*, 2015). These files currently do not work with MaxQuant (Tyanova *et al.*, 2016). Synthedia currently simulates post-translational carbamidomethylation of cysteine residues only. This constraint is due to the limitations in *in silico* prediction of complex peptide fragmentation for modified peptides with packages such as Prosit (Gessulat *et al.*, 2019).

## 3 Results

**Fig. 1.**
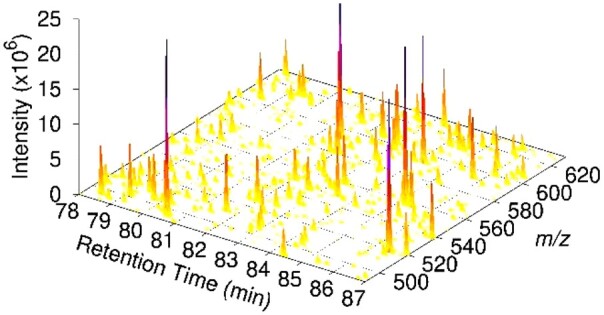
Subset of MS1 data for HeLa peptide ions generated with Synthedia

To demonstrate Synthedia, we performed LC-MS/MS analysis of a HeLa cell protein digest using a DDA method and created a Prosit spectral library from MaxQuant analysis results (see Supplementary information for details). The Prosit library was used to create a range of mzML file sets via simulation of the 35 768 peptide precursors identified. These can be obtained from the Synthedia web server and include sets of files with different gradient lengths, chromatographic peak widths, DIA window configurations and replicate structures. Example MS1 data are shown in Figure 1 and Supplementary Figure S1 and extracted ion chromatograms of the precursor and selected fragments for the precursor [AAAPGVEDEPLLR +2H]^2+^ are given in Supplementary Figure S2.

Simulations can be configured to generate mzML files that closely match the global characteristics of real data. For example, MaxQuant search results of HeLa analyses were compared to data generated with Synthedia and the distributions of chromatographic peak widths, precursor abundances and identifications were compared (Supplementary Figs S3–S5). The close similarity between real and synthetic data in each case indicates that global characteristics of simulated peptide ions can be made to match experimental results with an appropriate selection of input parameters.

**Fig. 2.**
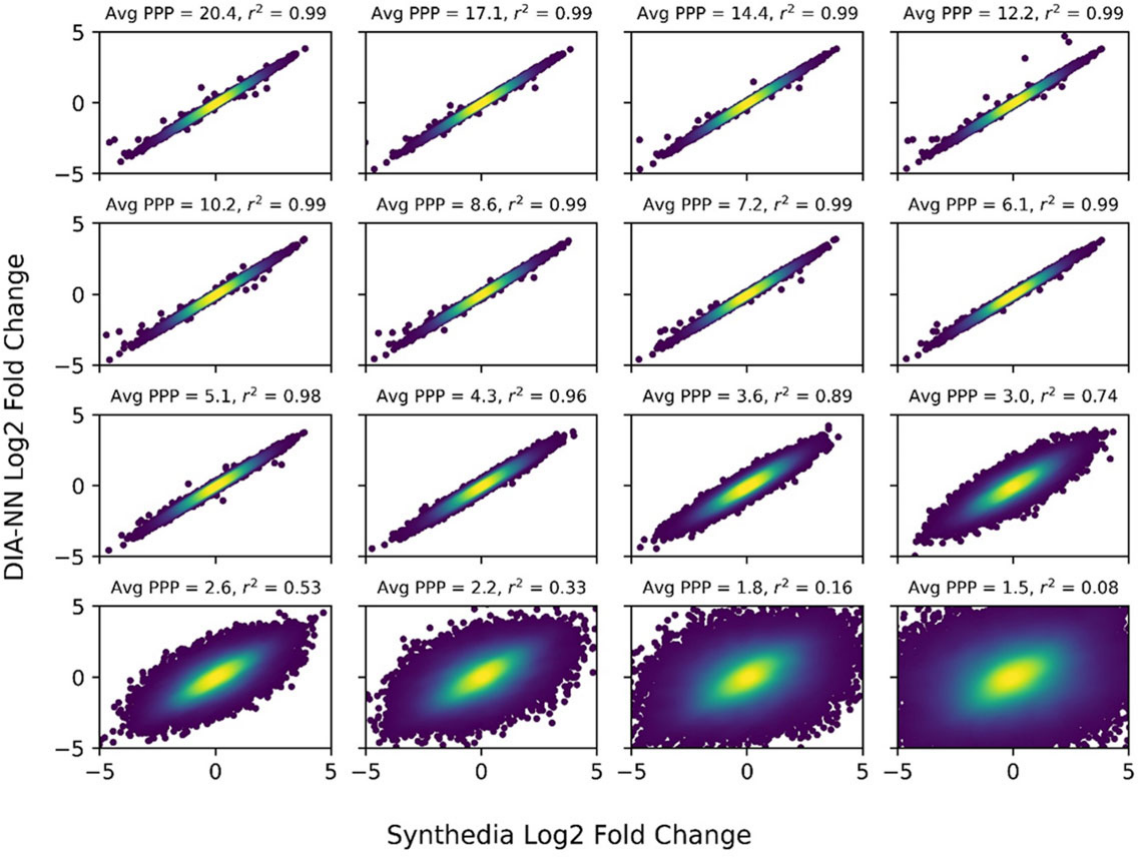
An example of using Synthedia to assess quantitative accuracy versus points across a chromatographic peak (machine cycle time). Comparison of peptide Log2 fold changes estimated by DIA-NN to the true values generated by Synthedia for a set of simulations of a two-group, three-replicate study analysed with different mass spectrum scan speeds. For each panel, ‘Avg PPP’ indicates the average number of mass spectral data points per chromatographic peak for that simulation and ‘r^2^’ indicates the coefficient of determination for a regression line y=mx+b. For ease of visualization, plots are restricted to the ranges of Log2(fold change) of −5 to +5

As an example of how Synthedia can be used to optimize data acquisition parameters, a series of two-group, three-replicate simulations were performed with varying mass spectral scan speeds. Increasing the time taken to acquire a single spectrum will decrease the number of spectra (and therefore data points) that can be acquired along the elution profile of a given peptide. Thus, these simulation sets contain variable numbers of data points per chromatographic peak. Simulated datasets were analysed with DIA-NN. The number of precursors identified by DIA-NN varied by scan speed but ranged between 22 484 and 32 615. The mean abundances of each group were calculated for precursors with no missing values and log2 fold changes generated by Synthedia and DIA-NN were compared (Fig. 2 and Supplementary Fig. S6). These data indicate deterioration of quantitative accuracy for data with fewer than *ca.* 6 data points per peak which is comparable to generally accepted practice (Bian *et al.*, 2020).

An attractive characteristic of simulations is that the number and identity of peptide precursors in the resultant files are exactly known. By comparison, biological factors such as gene silencing, post-transcriptional RNA processing and post-translational modification of proteins as well as sample handling and instrumental limitations make the true total complement of peptides present in a complex experimental protein digest near impossible to define. However, users should note that care is required when interpreting downstream identification rates from proteomic search software since some experimental effects are not modelled by Synthedia (or others), and the outputs are biased by the nature of the algorithms from which they are generated (Gatto *et al.*, 2016). For example, Synthedia models chromatographically overlapping peptides as a simple superposition of the expected precursor and fragment ions. In reality, simultaneous experimental detection of large numbers of precursors becomes limited by ion suppression and dynamic range constraints (Ang *et al.*, 2022; Annesley, 2003). As a result, mzML files produced by Synthedia are likely to more closely resemble true instrument data for longer gradients or simpler peptide mixtures. Nonetheless, ground-truth LC-MS/MS data simulated with Synthedia may be used to model instrumental acquisition methodologies and test downstream processing pipelines in the rapidly evolving field of DIA proteomics.

## Supplementary Material

vbac096_Supplementary_Data

## Data Availability

The source code for synthedia is publicly available and can be accessed at https://github.com/mgleeming/synthedia. A range of pre-generated mzML files can be obtained from our web server at https://synthedia.org/code or by request to the corresponding authors.
