# Supplementary material for: Simulation of mass spectrometry-based proteomics data with Synthedia
Source: Bioinform Adv. 2022 Dec 19;3(1):vbac096. doi: 10.1093/bioadv/vbac096 (PMC9825309; doi:10.1093/bioadv/vbac096)

Supplementary Information

Simulation of mass spectrometry-based proteomics data with Synthedia

Michael G. Leeming,^1^* Ching-Seng Ang,^1^ Shuai Nie,^1^ Swati Varshney,^1^ Nicholas A. Williamson^1^*

^1^Melbourne Mass Spectrometry and Proteomics Facility, Bio21 Molecular Science & Biotechnology Institute, The University of Melbourne, Australia.

*To whom correspondence should be addressed.

| **A/Prof. Nicholas A. Williamson**  Bio21 Molecular Science & Biotechnology Institute  The University of Melbourne  Melbourne, VIC, 3053  Australia  Tel: +61 3 8344 2206  E-Mail: nawill@unimelb.edu.au | **Dr. Michael G. Leeming**  Bio21 Molecular Science & Biotechnology Institute  The University of Melbourne  Melbourne, VIC, 3053  Australia  Tel: +61 3 8344 2540  E-Mail: leemingm@unimelb.edu.au |
| --- | --- |

**Contents**

[Supplementary Methods 3](#_Toc118817244)

[Figure S1 5](#_Toc118817245)

[Figure S2 6](#_Toc118817246)

[Figure S3 7](#_Toc118817247)

[Figure S4 8](#_Toc118817248)

[Figure S5 9](#_Toc118817249)

[Figure S6 10](#_Toc118817250)

[Table S1 11](#_Toc118817251)

[Table S2 16](#_Toc118817252)

[Table S3 17](#_Toc118817253)

[Synthedia web server tutorial 18](#_Toc118817254)

# Supplementary Methods

*Materials*

LC-MS grade Acetonitrile was purchased from Merck Millipore (MA, USA), Formic acid and Trifluoroacetic acid were from Pierce (Thermo Fisher Scientific, USA), and Pierce HeLa protein digest standard was obtained from Pierce (Thermo Fisher, USA) and high-purity water was produced with a Milli-Q filtration system.

*Mass Spectrometry*

LC-MS/MS was performed on an Eclipse Orbitrap mass spectrometer (Thermo Fisher, USA) equipped with a nano ESI interface coupled to an Ultimate 3000 nano HPLC (Thermo Fisher, USA). The LC system was equipped with an Acclaim Pepmap nano-trap column (Dinoex-C18, 100 Å, 75 μm X 2 cm) and an Acclaim Pepmap RSLC analytical column (Dinoex-C18, 100 Å, 75 μm X 50 cm). The tryptic peptides (1 µL, 100 ng) were injected into the enrichment column at an isocratic flow of 5 μL/min of 2% *v/v* CH_3_CN containing 0.05% *v/v* trifluoroacetic acid for 6 min, applied before the enrichment column was switched in-line with the analytical column. The eluents were 0.1% v/v formic acid (solvent A) in H_2_O and 100% *v/v* CH_3_CN in 0.1% *v/v* formic acid (solvent B. The gradient was at 300 nl min^-1^ from (i) 0–6 min, 3 % B; (ii) 6–95 min, 3–23 % B; (iii) 95-105 min, 23-40 % B; (iv) 105-110 min, 40-80 % B; (v) 110-115 min, 80–80 % B; (vi) 115-115.1 min, 80-3 % B; (vii) 115.1-125 min, 3-3 % B. The Eclipse Orbitrap mass spectrometer was operated in the data-dependent mode, whereby full MS^1^ spectra were acquired in a positive mode over the range of *m/z* 375-1500, with spray voltage at 1.9kV, source temperature at 275 °C, MS^1^ at 120,000 resolution, normalized AGC target of 100 % and maximum IT time of 22 ms. The top 3 second method was used and selecting peptide ions with charge states of ≥ 2-7 and intensity thresholds of ≥ 5E4 were isolated for MS/MS. The isolation window was set at 1.6 *m/z*, and precursors were fragmented using higher energy C-trap dissociation (HCD) at a normalised collision energy of 30, a resolution of 15,000, a normalized AGC target of 100% and automated IT time.

*Raw data analysis*

Mass spectrometry data were processed using MaxQuant version 2.0.1.0 (Tyanova et al., 2016) for the identification and quantification of peptides/proteins from the Human SwissProt database (May 2020). Fixed modifications of carbamidomethylation of cysteine as well as variable oxidation of methionine and protein N-terminal acetylation were permitted. Trypsin/P was set as the protease with a maximum of 2 missed cleavages. Protein and PSM false discovery rates (FDR) were both set at < 0.01.

*Construction of spectral library*

Peptide ions (that is, entities with unique sequence and charge) without variable PTMs were extracted from the ‘evidence.txt’ file produce via MaxQuant analysis. This resulted in a list of for the 36,212 precursors and MS/MS spectra were then predicted for these ions using Prosit. The collision energy was set to 30, ‘Prosit_2020_intensity_hcd’ was used as the intensity prediction model ‘Prosit_2019_irt’ was set for iRT prediction and ‘Generic text’ output was requested.

*Data points per peak quantitative accuracy survey*

The prosit library generated above was used with Synthedia to simulate a series of experiments wherein different numbers of data points were acquired for chromatographic peaks. This was achieved by setting the MS^1^ scan speed to a low value of 0.01 s and systematically varying the MS^2^ scan speed. Data were simulated in centroid mode for both MS^1^ and MS^2^ levels, the DIA window width was set to 14 Th and the MS1 acquisition range was *m/z* 350-1400. Two treatment groups were simulated with three replicates per group. All other parameters were as default.

The 6 mzML files produced by each simulation were analysed using DIA-NN (version 1.8) against a spectral library generated by the in-built *in silico* prediction algorithm from a FASTA file containing the sequences for proteins identified by MaxQaunt analysis of DDA mass spectrometry data above. These data were analysed with MS^1^ and MS^2^ mass accuracies fixed at 10 ppm. The scan windows were fixed at the maximum number of data points per peak for any peptide for a given set of Synthedia input files The match between runs feature was activated and all other parameters were as default.

The precursor matrices produced by DIA-NN and the peptide tables written by Synthedia were used for construction of accuracy plots. Quantitative values were averaged for samples of each treatment group and then the ratio of mean group abundances was Log2 transformed. Peptides with any missing or ‘0’ values were excluded from the analysis.

*Comparison between real and simulated data*

Synthedia simulations were conducted using the ‘*evidence.txt*’ and ‘*msms.txt*’ files produced by the MaxQuant search above. Both, MS1 and MS2 spectra were simulated in centroid mode, the isolation window was set to 14 Th, and the rt_peak_fwhm_distribution mean, standard deviation and shape factor (‘emg_k’) were set to 4.25, 1.4 and 2.5 respectively. The run length was set to 125 and no retention time buffer was applied.

Density plots for chromatographic peak width and precursor abundance, as well as scatter plots of cumulative peptide identifications vs. retention time were produced from data contained in the ‘*evidence.txt*’ and ‘*peptide_table.tsv*’ files produced by MaxQuant and Synthedia respectively. Plots were generated using Seaborn (v0.11.1) in Python.


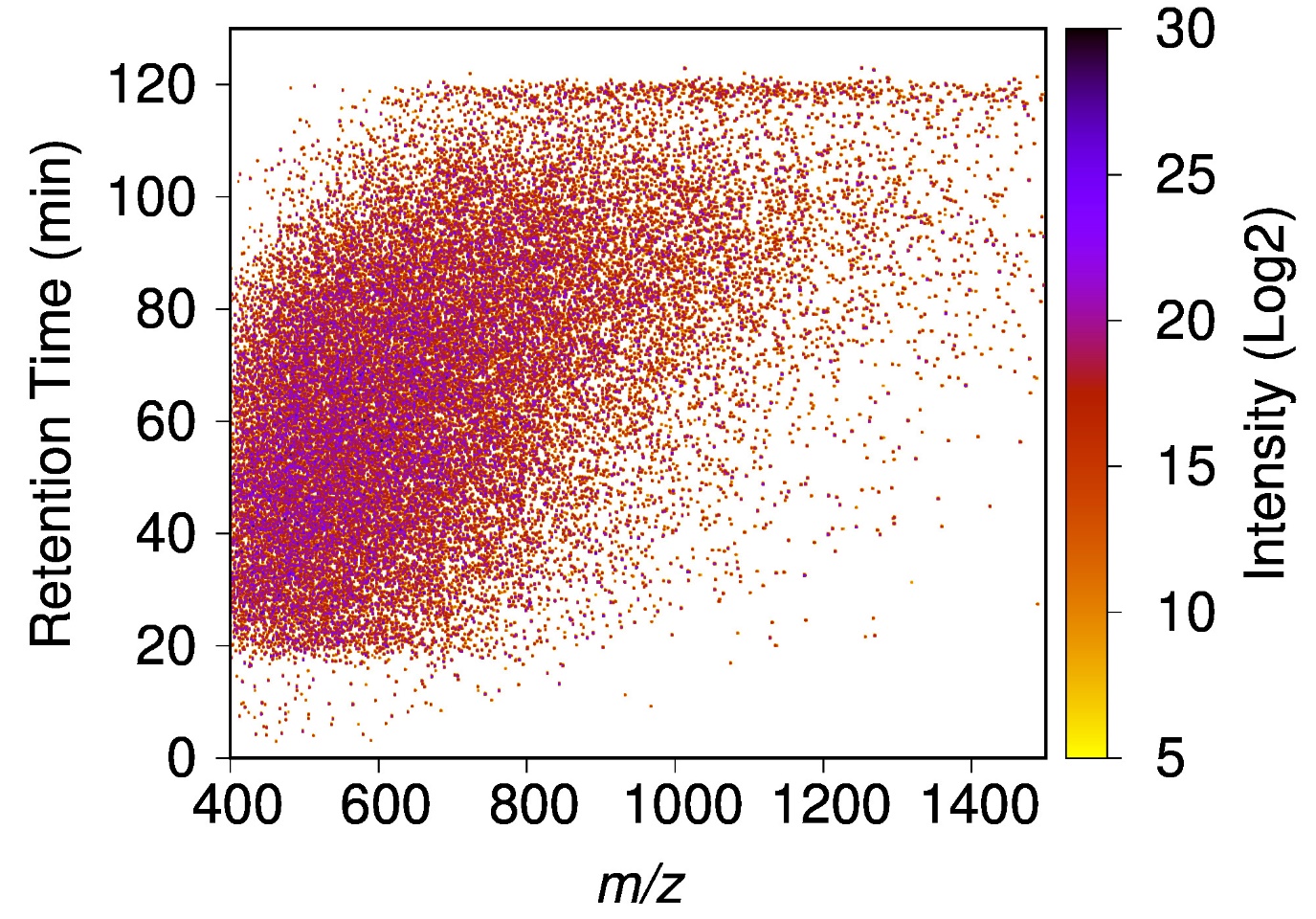


Figure S1**.** Heatmap of Log2-transformed MS^1^ signal intensities for 35,768 peptide precursors simulated with Synthedia.

**
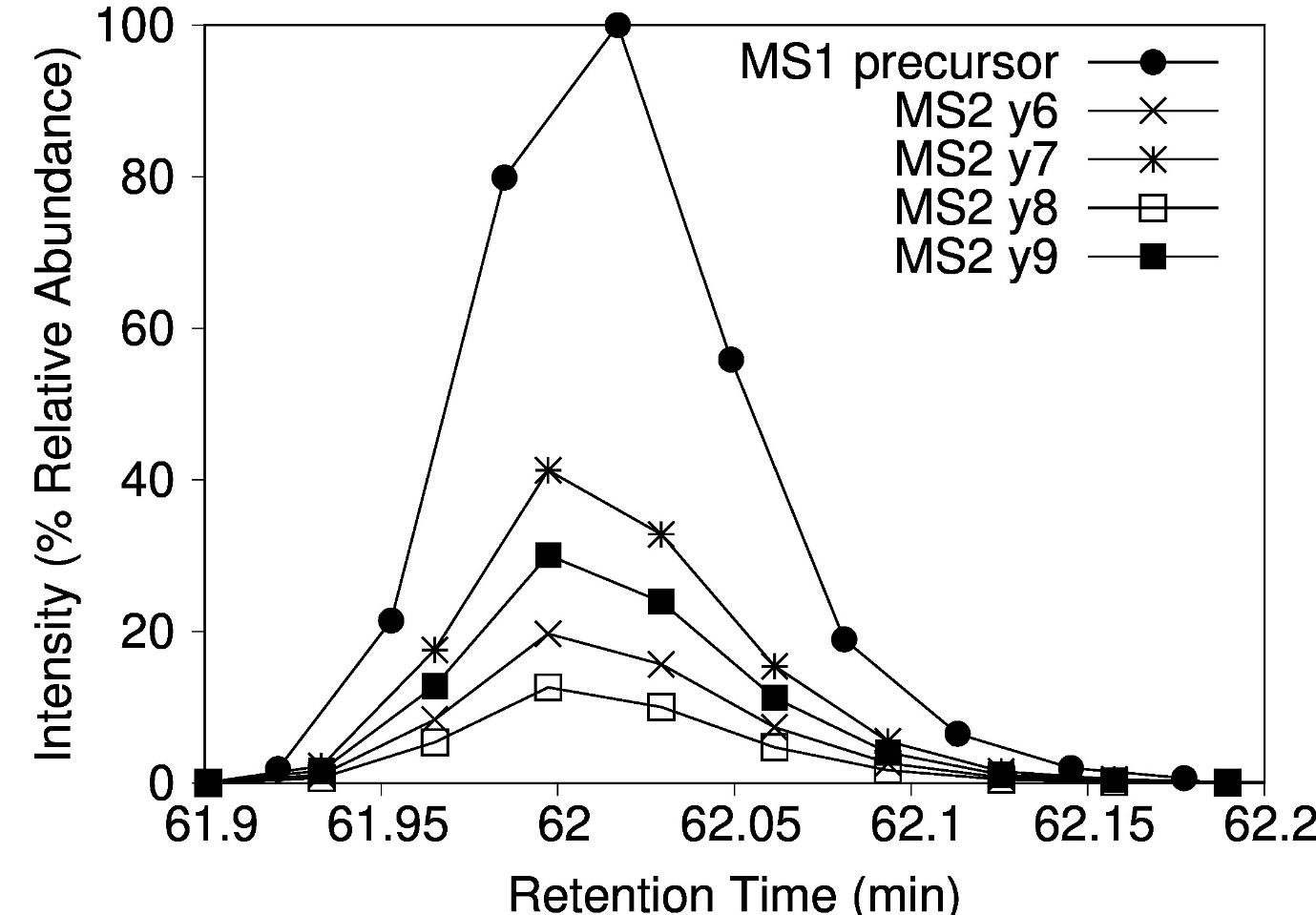
**

Figure S2**.** Extracted ion chromatogram of *m/z* 669.35 for the peptide ion [AAAPGVEDEPLLR +2H]^2+^ and selected fragmentation products. The intensity scale is normalised to the maximum MS1 intensity.


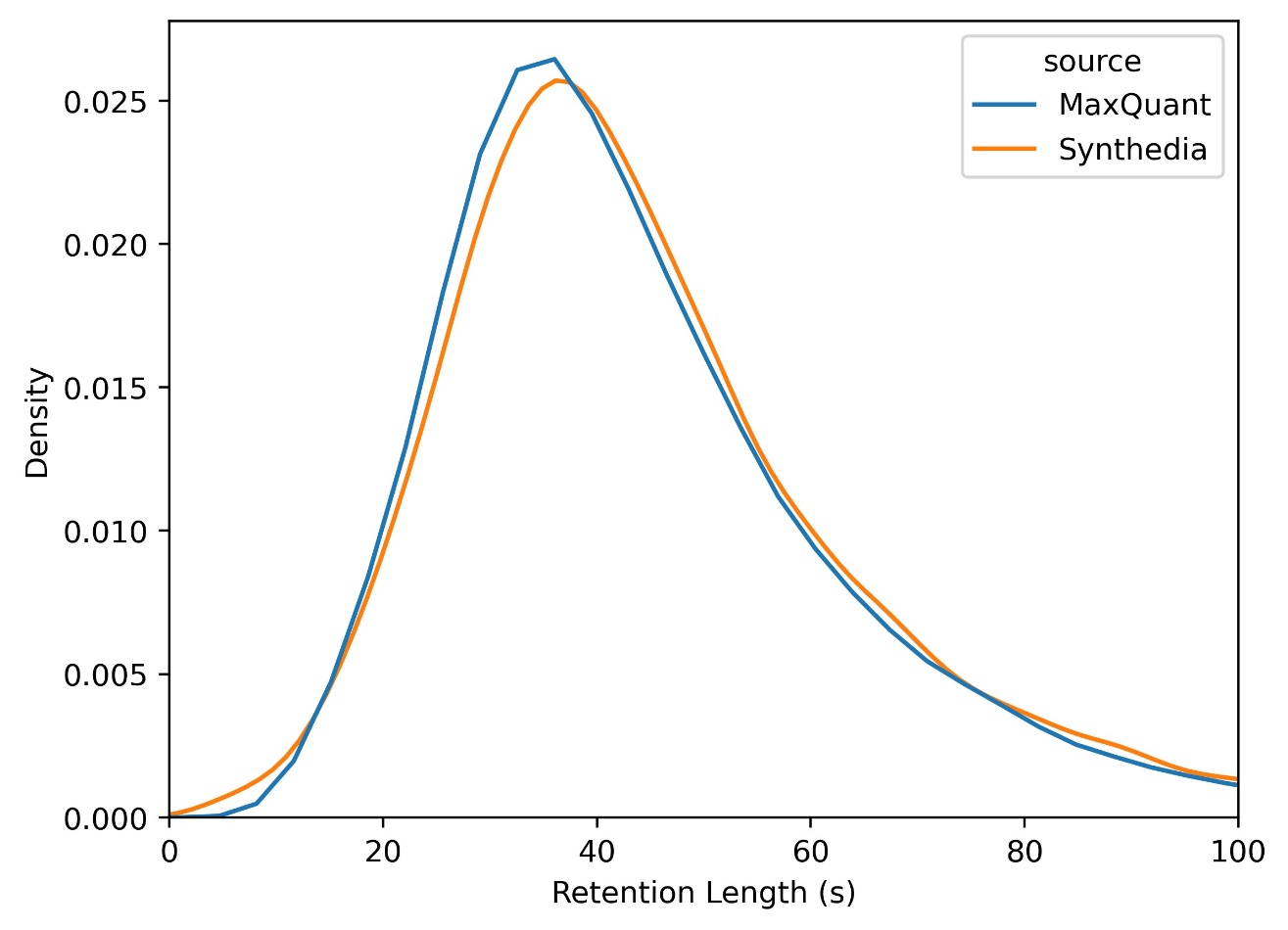


Figure S3**.** Kernel density estimate plot of chromatographic peak widths. The blue trace represents values obtained from MaxQuant analysis of experimentally analysed HeLa peptides. The orange trace represents values produced by Synthedia following simulation of these same peptides. Note that Retention Length values (x-axis) are the total retention time of a peptide (*i.e.* time last observed – time first observed).


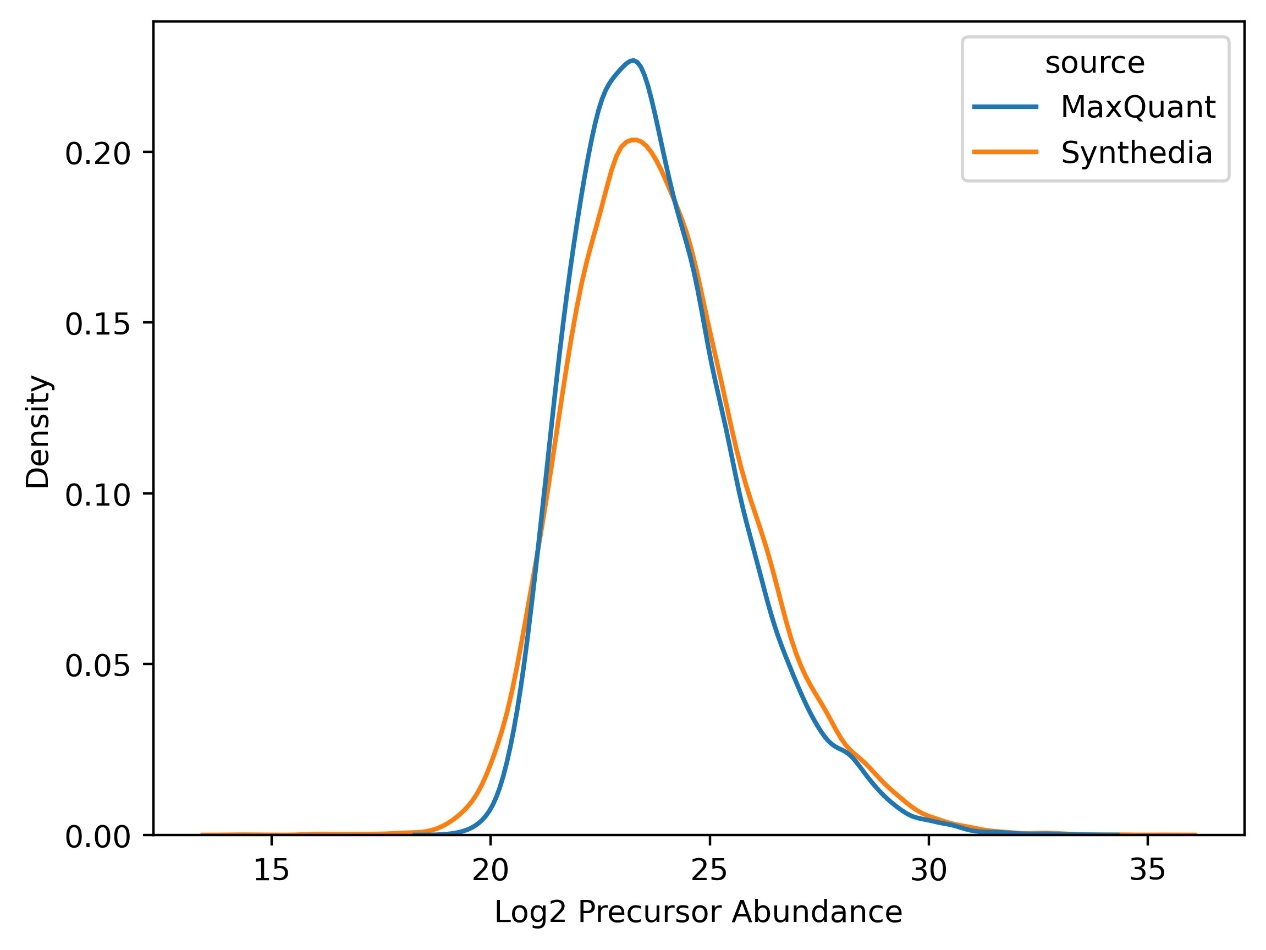


Figure S4**.** Kernel density estimate plot of Log2 precursor ion abundances. The blue trace represents values obtained from MaxQuant analysis of experimentally analysed HeLa peptides. The orange trace represents values produced by Synthedia following simulation of these same peptides.


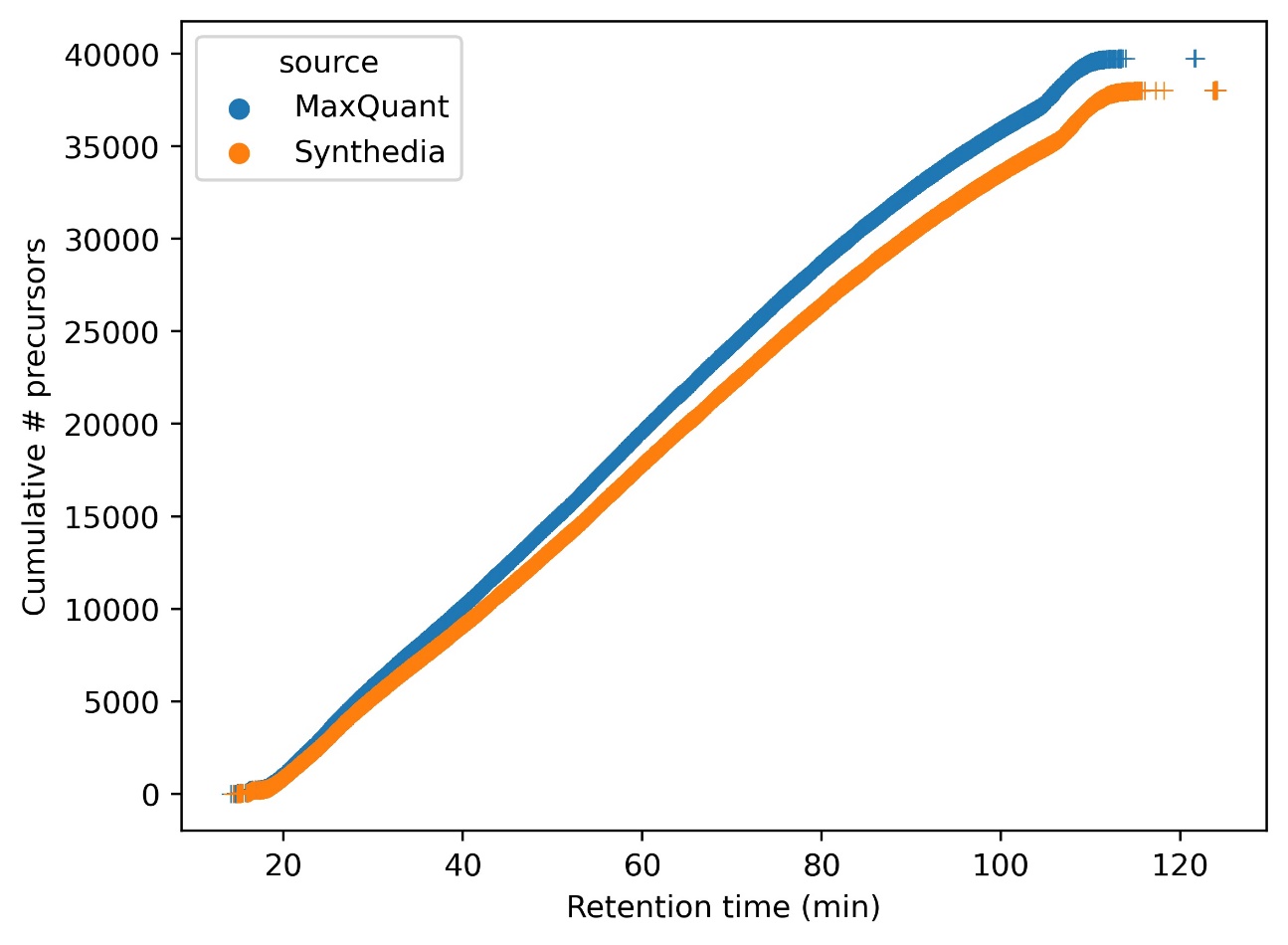


Figure S5**.** Scatterplot of the number of precursors simulated and identified as a function of retention time. The blue trace represents values obtained from MaxQuant analysis of experimentally analysed HeLa peptides. The orange trace represents values produced by Synthedia following simulation of these same peptides.


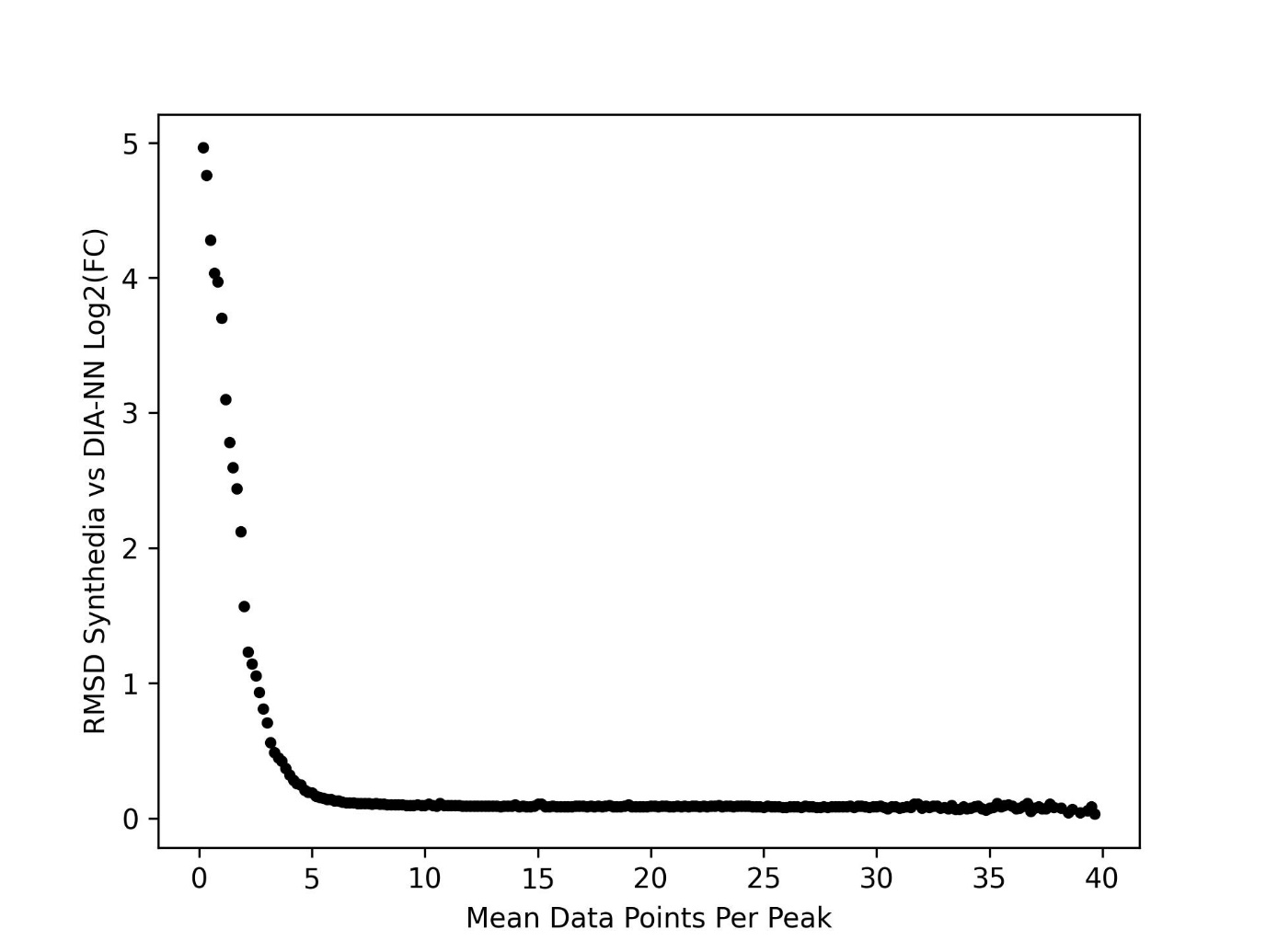


Figure S6**.** Root-mean-square deviation between Synthedia and DIA-NN Log2 ratios for the data presented in Figure S3 as a function of mean data points per chromatographic peak.

Table S1**.** List of Synthedia input parameters.

| **Parameter** | **Description** |
| --- | --- |
|  |  |
| **Input/Output** |  |
| mq_txt_dir | Path to MaxQuat "txt" directory. |
| prosit | Path to prosit prediction library. |
| prosit_peptide_abundance_model | The model from which log2 abundance values are drawn to generate abundances for prosit precursors. Can be "gaussian", "exponentially_modified_gaussian" or "cauchy". Not used for MaxQuant input types. |
| prosit_peptide_abundance_mean | Mean log2 abundance used to simulate peptide abundances for prosit input types. Not used for MaxQuat input types. |
| prosit_peptide_abundance_stdev | Standard deviation of gaussian used to simulate peptide abundances for prosit input types. Not used for MaxQuant input types. |
| prosit_peptide_abundance_emg_k | Shape factor for exponentially modified gaussian from which peptide abundances are drawn for prosit input types. Must be greater than 0. Increasing this value results in a more heavily tailed distribution resulting in more peptides higher intensities. Not used for MaxQuant input types |
| acquisition_schema | Path to file defining MS2 acquisition schema. |
| use_existing_peptide_file | Path to an existin peptide file which will be used. |
| out_dir | Output directory where results should be written. |
| output_label | Prefix for output files. |
| config | Path to *.yaml config file. |
| silent | Do not print logging output to terminal |
| write_params | Write parameters to yaml file and exit. |
|  |  |
| **Filtering** |  |
| mq_pep_threshold | For MaxQuant input data, use only peptides with a Posterior Error Probability (PEP) less than this value |
| filterTerm | Terms used to filter input maxquant lists to remove unwanted targets. For example contaminant protein can be removed by specifying "--filterTerm CON_". Multiple filters can be applied. For example "--filterTerm CON_ --filterTerm REV_". Filters are used only for MaxQuant input types (no effect for Prosit) and are applied to the "Proteins" column of the evidence.txt table. |
|  |  |
| **Processing** |  |
| num_processors | Number of cores to use in constructing mzML files. Defaults to all available cores |
|  |  |
| **Instrument Parameters** |  |
| ms1_min_mz | Minimum m/z at MS1 level. |
| ms1_max_mz | Maximum m/z at MS1 level. |
| ms2_min_mz | Minimum m/z at MS2 level. |
| ms2_max_mz | Maximum m/z at MS2 level. |
| ms1_resolution | Mass spectral resolution at MS1 level. |
| ms2_resolution | Mass spectral resolution at MS2 level. |
| ms1_scan_duration | Time in seconds taken to record an MS1 scan. |
| ms2_scan_duration | Time in seconds taken to record an MS2 scan. |
| isolation_window | Length of DIA window in m/z. |
| resolution_at | m/z value at which resolution is defined. |
| n_points_gt_fwhm | Number of MS data points greater than the peak FWHM. Increasing this number means each mass spectral peak will be described by more data points but will also slow processing time and increase file size. |
| esi_instability | Simulates imperfection in chromatographic peaks by applying a randomly intensity scaling factor to adjacent scans. A value of 0 indicates no randomness. A value of 100 indicates high spray instability. |
| ms1_ppm_error_mean | The mean value of a Gaussian distribution from which PPM errors for MS1 precursors will be drawn. This value can be negative. Setting both ms1_ppm_error_mean, and ms1_ppm_error_stdev to 0 equates to perfect mass accuracy. |
| ms1_ppm_error_stdev | The standard deviation of a Gaussian distribution from which PPM errors for MS1 precursors will be drawn. Setting both ms1_ppm_error_mean and ms1_ppm_error_stdev to 0 equates to perfect mass accuracy. |
| ms2_ppm_error_mean | The mean value of a Gaussian distribution from which PPM errors for MS2 fragments will be drawn. This value can be negative. Setting both ms1_ppm_error_mean, and ms1_ppm_error_stdev to 0 equates to perfect mass accuracy. |
| ms2_ppm_error_stdev | The standard deviation of a Gaussian distribution from which PPM errors for MS2 fragments will be drawn. Setting both ms1_ppm_error_mean and ms1_ppm_error_stdev to 0 equates to perfect mass accuracy. |
|  |  |
| **Chromatography** |  |
| rt_peak_fwhm_distribution_model | The model used to simulate chromatographic peaks. Can be "gaussian", "exponentially_modified_gaussian" or "cauchy" |
| rt_peak_fwhm_distribution_mean | Chromatographic peak full with at half maximum intehsity in seconds. If --rt_peak_fwhm_stdev is non-zero, this value is the mean of a Gaussian distribution from which FWHMs for individual peptides are drawn |
| rt_peak_fwhm_distribution_stdev | Standard deviation of a Gaussian distribution from which chromatographic peak full with at half maximum intehsity are drawn for indifivual peptides. |
| rt_peak_fwhm_distribution_emg_k | Shape factor for exponentially modified gaussian from which peak FWHMs are drawn. Must be greater than 0. Increasing this value results in a more heavily tailed distribution resulting in more peptides with broad chromatographic profiles |
| min_rt_peak_fwhm | Minimum chromatographic peak full with at half maximum intehsity in seconds. |
| original_run_length | Length in minutes of original data file. If not given, this will be determined by taking the difference between the minimum and maximum peptide retention times. If set to "0", the retention time range will be automatically detected from the input data. |
| new_run_length | Length in minutes of new data file. If set to "0", the retention time range of the input data will be used. |
| rt_buffer | Time (in minutes) that should be appended to the beginning and end of the retention time range of a set of input peptides. This helps ensure that peptides at the boundaries of the elution window are simulated completely |
| rt_instability | Introduces an instability in retentention time values for the same peptide when a multi-group or multi-sample simulation is conducted. The value is the maximum number of seconds by which peptide retention times will differ |
|  |  |
| **Simulation** |  |
| ms1_min_peak_intensity | Peptide elution profiles are simulated as gaussian peaks. This value sets the minimum gaussian curve intensitiy for a peptide to be simulated in MS1 spectra. |
| ms2_min_peak_intensity | Peptide elution profiles are simulated as gaussian peaks. This value sets the minimum gaussian curve intensitiy for a peptide to be simulated in MS2 spectra. |
| centroid_ms1 | If given, simulated MS1 mass spectra will be centroided. Otherwise, profile data will be written. |
| centroid_ms2 | If given, simulated MS2 mass spectra will be centroided. Otherwise, profile data will be written. |
| write_empty_spectra | Write empty mass sepctra to the output data file |
| mz_peak_model | The model used to simulate mass spectral peaks. Can be "gaussian", "exponentially_modified_gaussian" or "cauchy" |
| rt_peak_model | The model used to simulate chromatographic peaks. Can be "gaussian", "exponentially_modified_gaussian" or "cauchy" |
| mz_emg_k | Shape factor for exponentially modified gaussian in the mass spectral domain. Must be greater than 0. Increasing K results in more heavily tailed mass spectral peaks. This parameter is inactive unless --mz_peak_model is not set to exponentially_modified_gaussian. |
| rt_emg_k | Shape factor for exponentially modified gaussian in the retention time domain. Must be greater than 0. Increasing K results in more heavily tailed chromatographic peaks. This parameter is inactive unless --rt_peak_model is not set to exponentially_modified_gaussian. |
| prob_missing_in_sample | Probability (0-100) that a peptide is missing in any given sample |
| prob_missing_in_group | Probability (0-100) that a peptide is missing in an entire group |
| no_isotopes | Disable simulation of non-monoisotopic ions. Can be used to increase simulation speed when optimising parameters. |
|  |  |
| **Plotting** |  |
| tic | Plot TIC for the generated mzML file. |
| schema | Plot acquisition schema. |
| all | Plot all graphics. |
|  |  |
| **Grouping and Quantitation** |  |
| n_groups | Number of treatment groups to simulate. |
| samples_per_group | Number of individual samples to simulate per treatment group. |
| between_group_stdev | Standard deviation of a normal distribution from which group means will be drawn. |
| within_group_stdev | Standard deviation of a normal distribution from which within group samples will be drawn. |
|  |  |
| **Decoys** |  |
| decoy_msp_file | Path to MSP file. Note - must include retention times. |
| num_decoys | Number of decoy peaks to simulate |
| simulate_top_n_decoy_fragments | Simulate n most intense fragments of the decoy compound. |
| decoy_abundance_mean | Mean log2 abundance used to simulate decoy ion abundances. |
| decoy_abundance_stdev | Standard deviation of gaussian used to simulate decoy ion abundances. |
|  |  |
| **Preview** |  |
| preview | Simulate a single peptide without a Prosit/MaxQuant file. Useful for testing parameter sets. |
| preview_sequence | Primary sequence of peptide to preview. |
| preview_charge | Charge of intact peptide to preview. |
| preview_abundance | Abundance of peptide to simulate |

Table S2**.** Example of a custom DIA acquisition schema file for 14 Th windows offset by 50%. For brevity, only windows in the region of *m/z* 400 to *m/z* 600 are shown here.

| **ms_level** | **scan_duration**  **in_seconds** | **isolation_window**  **lower_mz** | **isolation_window**  **upper_mz** |
| --- | --- | --- | --- |
| 1 | 0.2 | 0 | 0 |
| 2 | 0.04 | 400 | 414 |
| 2 | 0.04 | 414 | 428 |
| 2 | 0.04 | 428 | 442 |
| 2 | 0.04 | 442 | 456 |
| 2 | 0.04 | 456 | 470 |
| 2 | 0.04 | 470 | 484 |
| 2 | 0.04 | 484 | 498 |
| 2 | 0.04 | 498 | 512 |
| 2 | 0.04 | 512 | 526 |
| 2 | 0.04 | 526 | 540 |
| 2 | 0.04 | 540 | 554 |
| 2 | 0.04 | 554 | 568 |
| 2 | 0.04 | 568 | 582 |
| 2 | 0.04 | 582 | 596 |
| 1 | 0.2 | 0 | 0 |
| 2 | 0.04 | 407 | 421 |
| 2 | 0.04 | 421 | 435 |
| 2 | 0.04 | 435 | 449 |
| 2 | 0.04 | 449 | 463 |
| 2 | 0.04 | 463 | 477 |
| 2 | 0.04 | 477 | 491 |
| 2 | 0.04 | 491 | 505 |
| 2 | 0.04 | 505 | 519 |
| 2 | 0.04 | 519 | 533 |
| 2 | 0.04 | 533 | 547 |
| 2 | 0.04 | 547 | 561 |
| 2 | 0.04 | 561 | 575 |
| 2 | 0.04 | 575 | 589 |
| 2 | 0.04 | 589 | 603 |

Table S3**.** Run time examples for simulations performed with Synthedia using different inputs and parameters. Simulations were performed using a Dell Inc. Precision 5510 Laptop with Intel® Core™ i7-6820HQ CPU @ 2.70GHz × 8 and 31 GB RAM. In each case, a single mzML file was generated (*i.e.* a multi-group comparison simulation was not conducted).

| **Cores** | **# Precursors** | **Centroid MS1** | **Centroid MS2** | **Precursor**  **modelling time (min)** | **mzML assembly time (min)** | **Total run time (min)** |
| --- | --- | --- | --- | --- | --- | --- |
| 8 | 7178 | TRUE | TRUE | 1.10 | 0.25 | 1.35 |
| 8 | 14370 | TRUE | TRUE | 2.17 | 0.43 | 2.6 |
| 8 | 21716 | TRUE | TRUE | 3.28 | 0.60 | 3.9 |
| 8 | 28614 | TRUE | TRUE | 4.43 | 0.77 | 5.23 |
| 8 | 35768 | TRUE | TRUE | 5.60 | 0.95 | 6.58 |
| 1 | 35768 | TRUE | TRUE | 15.23 | 0.93 | 16.2 |
| 2 | 35768 | TRUE | TRUE | 9.28 | 0.95 | 10.27 |
| 3 | 35768 | TRUE | TRUE | 6.85 | 0.95 | 7.83 |
| 4 | 35768 | TRUE | TRUE | 5.60 | 0.95 | 6.57 |
| 5 | 35768 | TRUE | TRUE | 5.98 | 0.95 | 6.97 |
| 6 | 35768 | TRUE | TRUE | 5.63 | 0.95 | 6.6 |
| 7 | 35768 | TRUE | TRUE | 5.43 | 0.95 | 6.42 |
| 8 | 35768 | TRUE | TRUE | 5.62 | 0.93 | 6.6 |
| 1 | 35768 | FALSE | FALSE | 15.2 | 46.08 | 61.32 |
| 2 | 35768 | FALSE | FALSE | 9.37 | 46.02 | 55.43 |
| 3 | 35768 | FALSE | FALSE | 6.90 | 45.45 | 52.38 |
| 4 | 35768 | FALSE | FALSE | 5.57 | 45.67 | 51.27 |
| 5 | 35768 | FALSE | FALSE | 6.08 | 45.45 | 51.57 |
| 6 | 35768 | FALSE | FALSE | 5.43 | 45.73 | 51.2 |
| 7 | 35768 | FALSE | FALSE | 5.42 | 45.47 | 50.92 |
| 8 | 35768 | FALSE | FALSE | 5.70 | 45.42 | 51.17 |

# Synthedia web server tutorial

1. Navigate to <https://www.synthedia.org/>
2. To begin a new simulation, click ‘Create’ in the navigation sidebar.


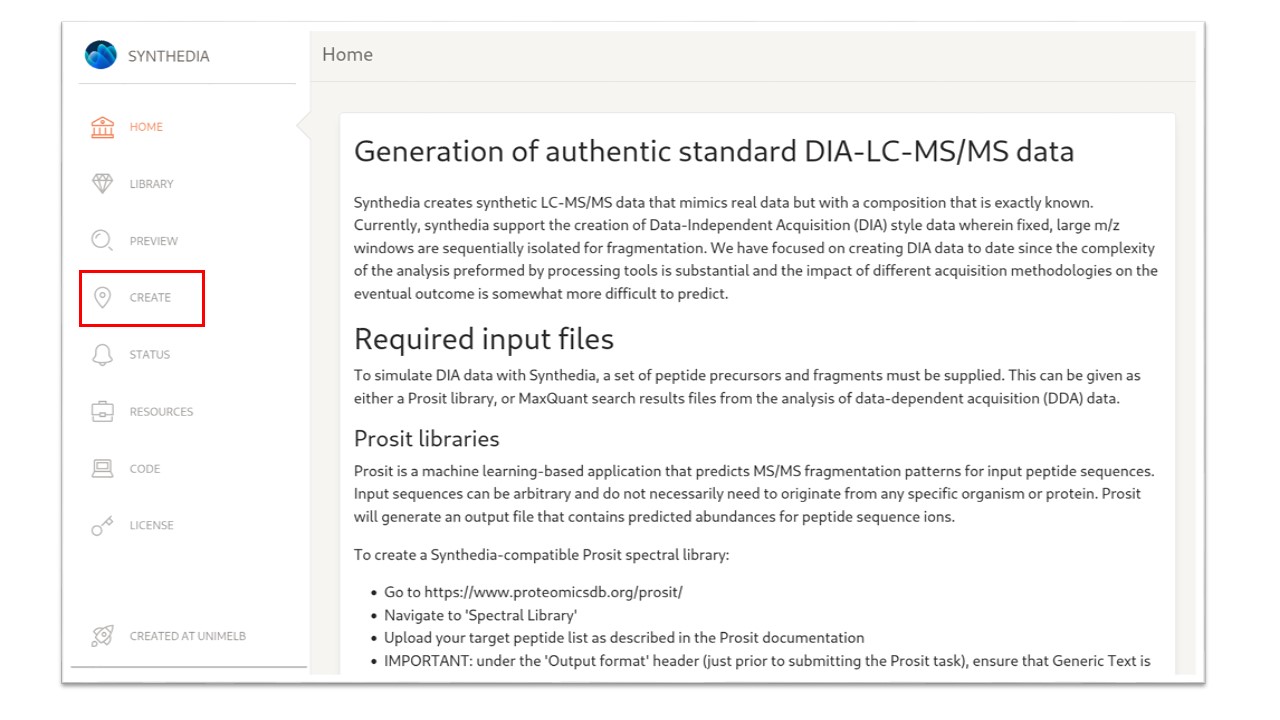


1. Provide input files containing the ions that you wish to simulate. If the ‘MaxQuant’ input option is selected, upload both the ‘evidence.txt’ and ‘msms.txt’ files located in the ‘txt’ output directory. For ‘Prosit’, upload the single ‘.csv’ library file produced by Prosit.


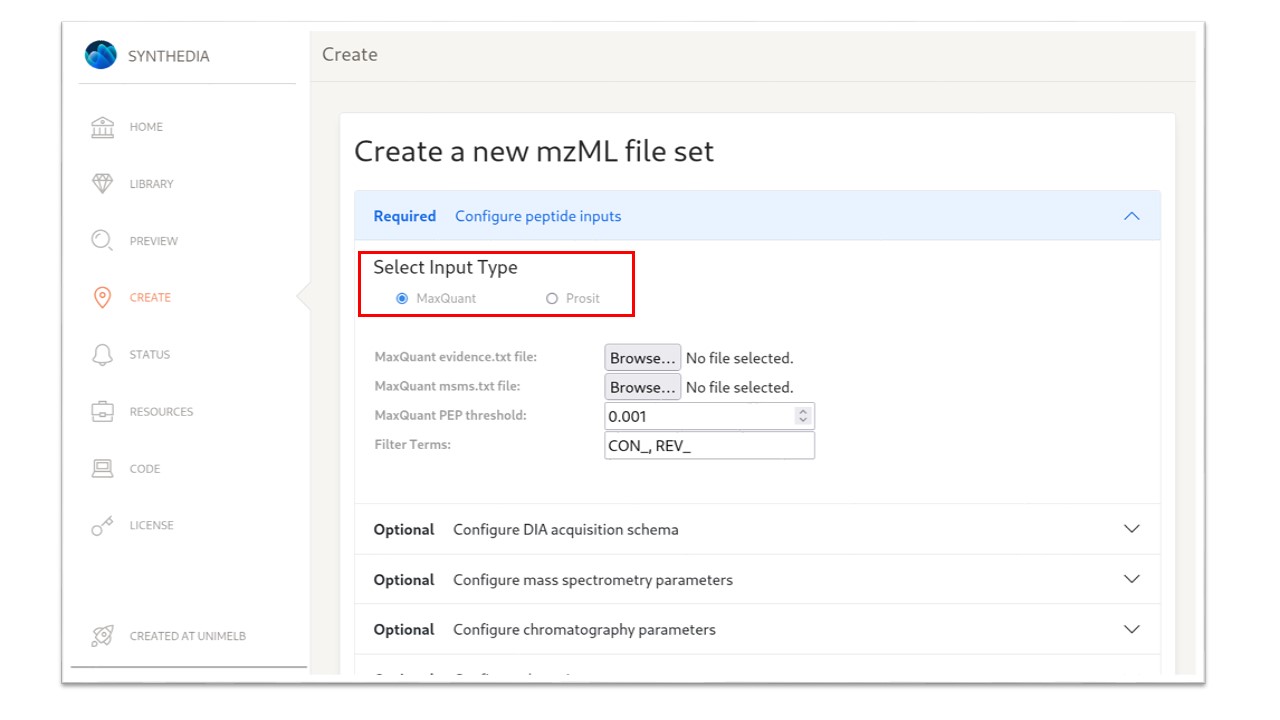


1. Step through each category of input parameters and set values appropriate for your simulation.


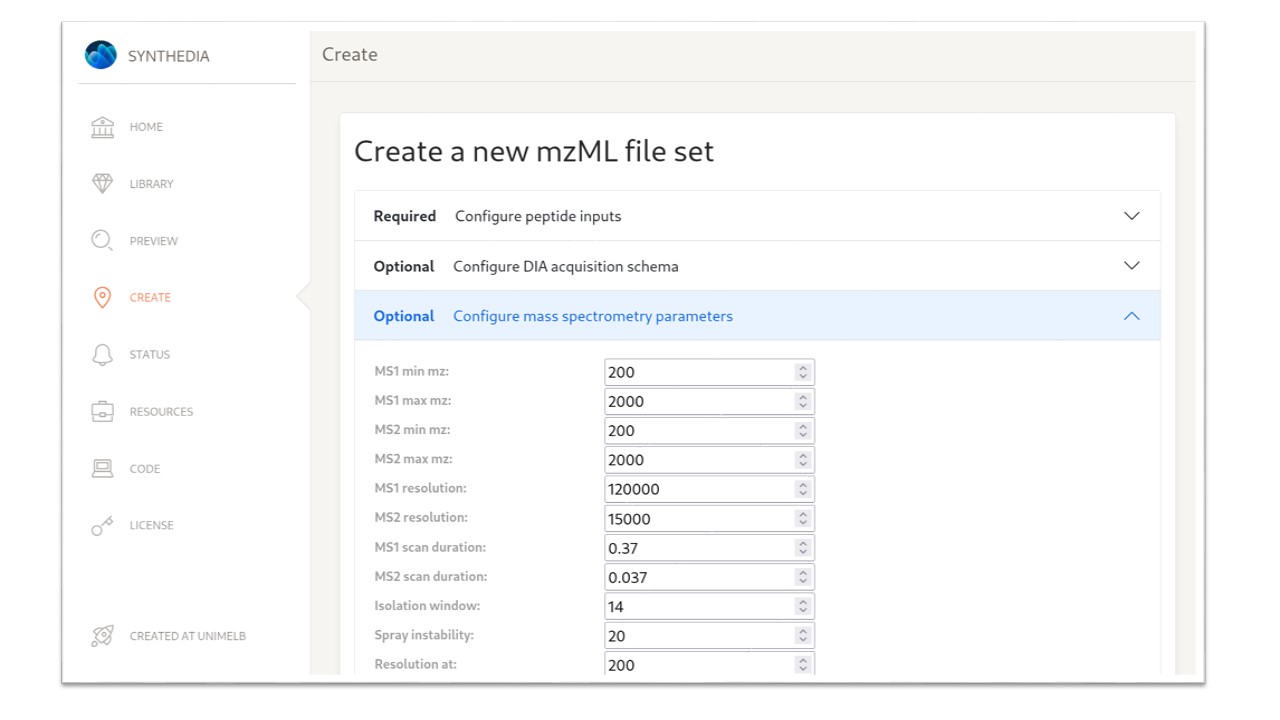


1. Hover your mouse over any of the input fields to see a popup containing a descript of the parameter.


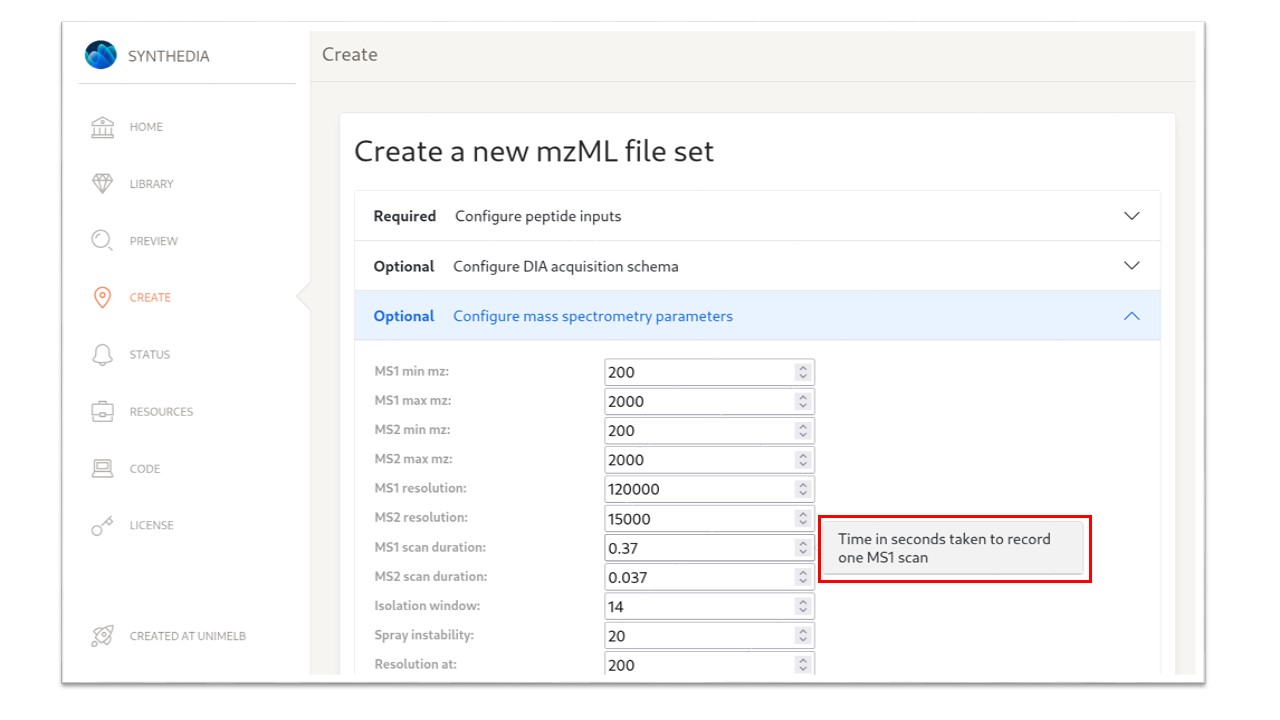


1. Once you are happy with all parameters, click ‘submit’.


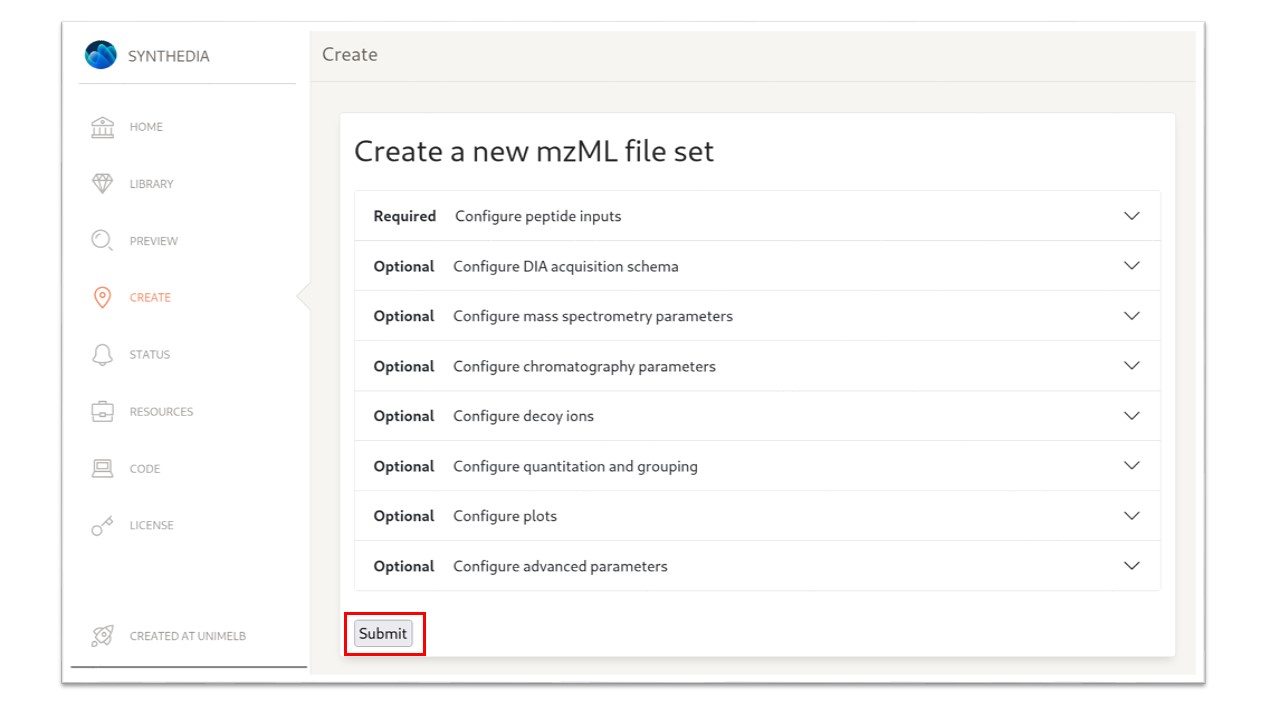


1. Following upload of your input files, you will be redirected to the ‘Status’ page where you can monitor the progress of your task. An ID code is generated that is unique to your simulation. You can bookmark this page in your internet browser, or retain this ID to return to your dataset at a later time.
2. Click ‘submit’ to check the status of your simulation.


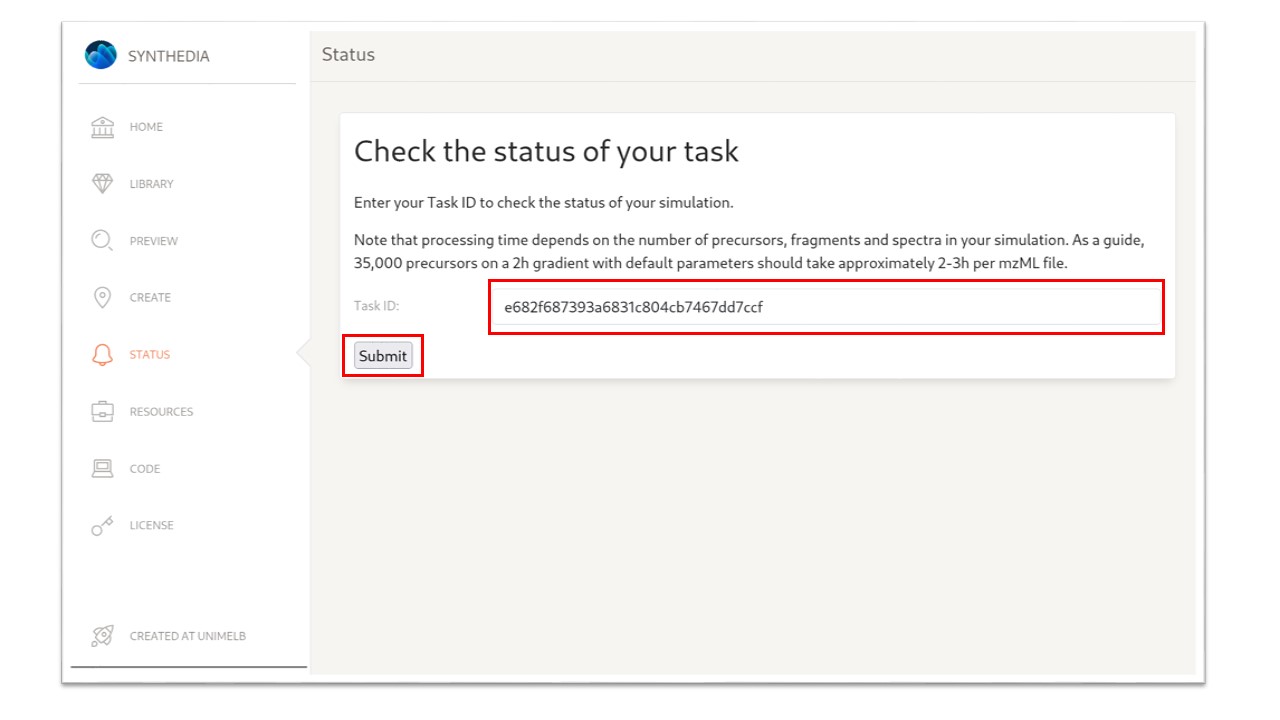


1. Here, we can see that the simulation is still in progress. The simulation log file is given in the box at the end of the page.


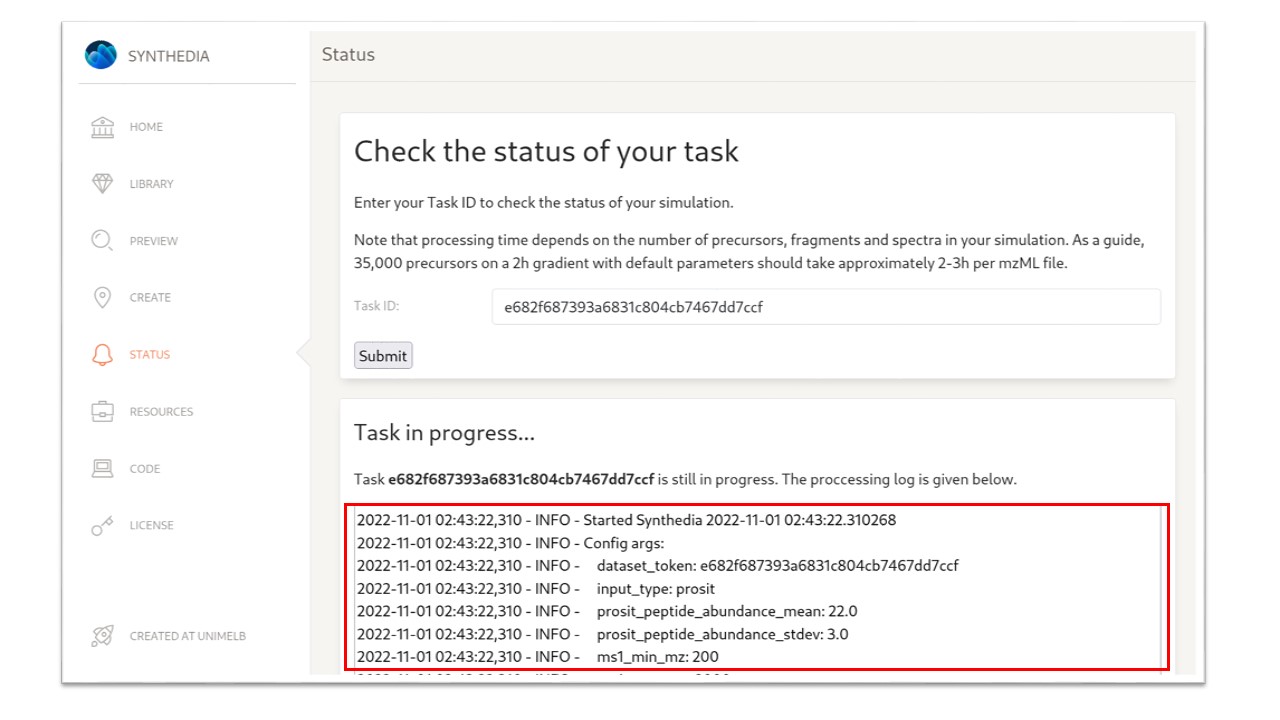


1. Clicking ‘submit’ again refreshes the page. Our simulation is now done and we can download the files produced.


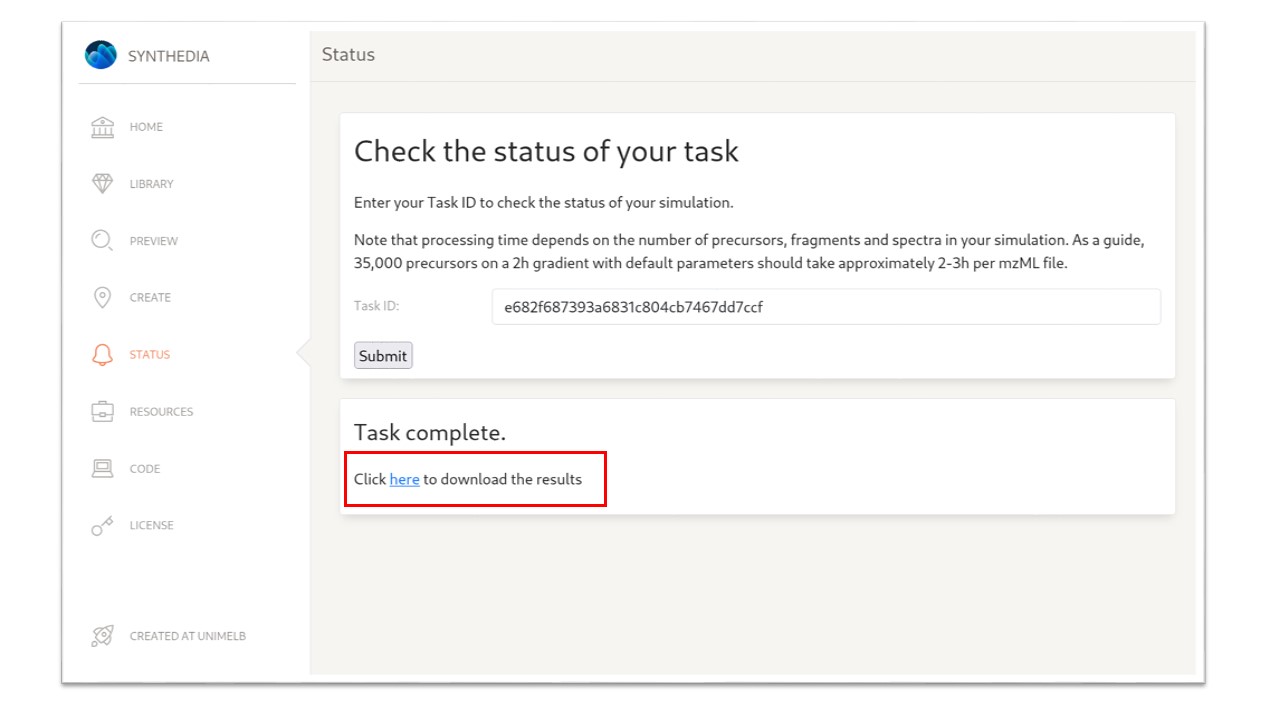


1. The download is a zip archive containing the mzML file(s) produced, the table of peptide ions simulated, the processing log and input arguments, and any images requrested.


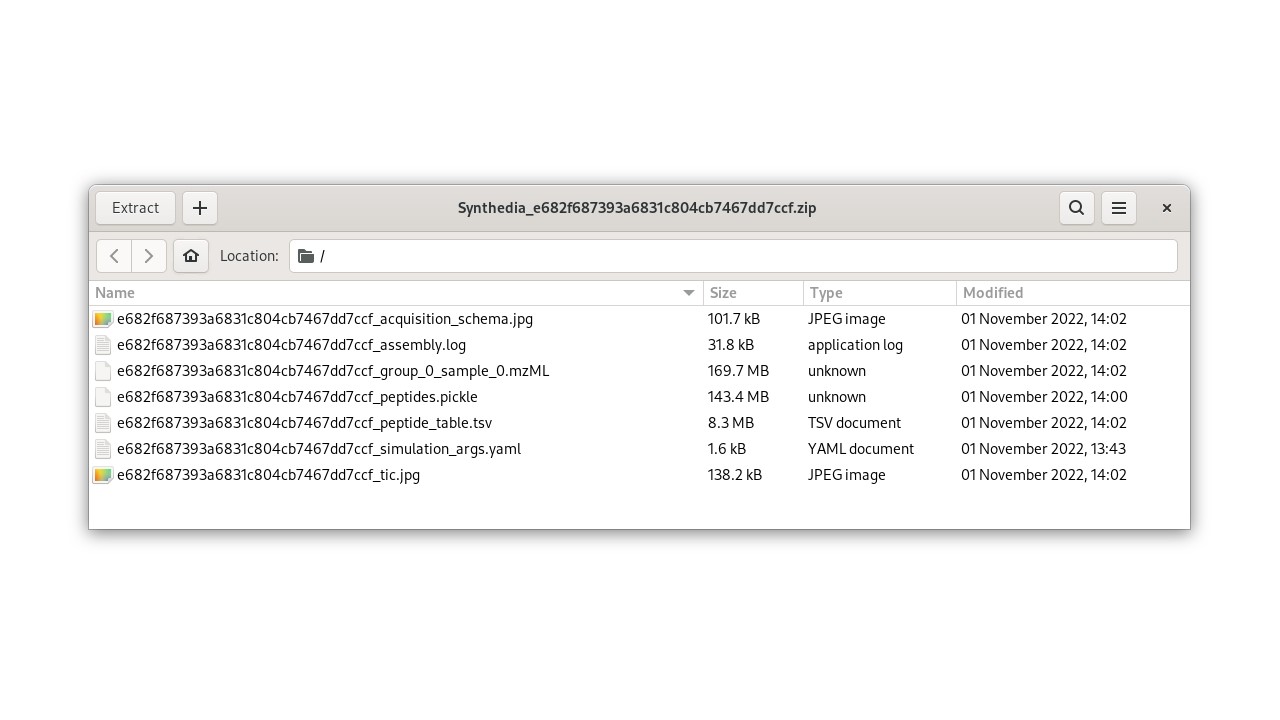


1. The ‘Preview’ tool can be used to rapidly visualise the impact of changing some key simulation parameters. Here, a single peptide is simulated in a given charge state and abundance. Once you have set the parameters as appropriate, click ‘Submit’.


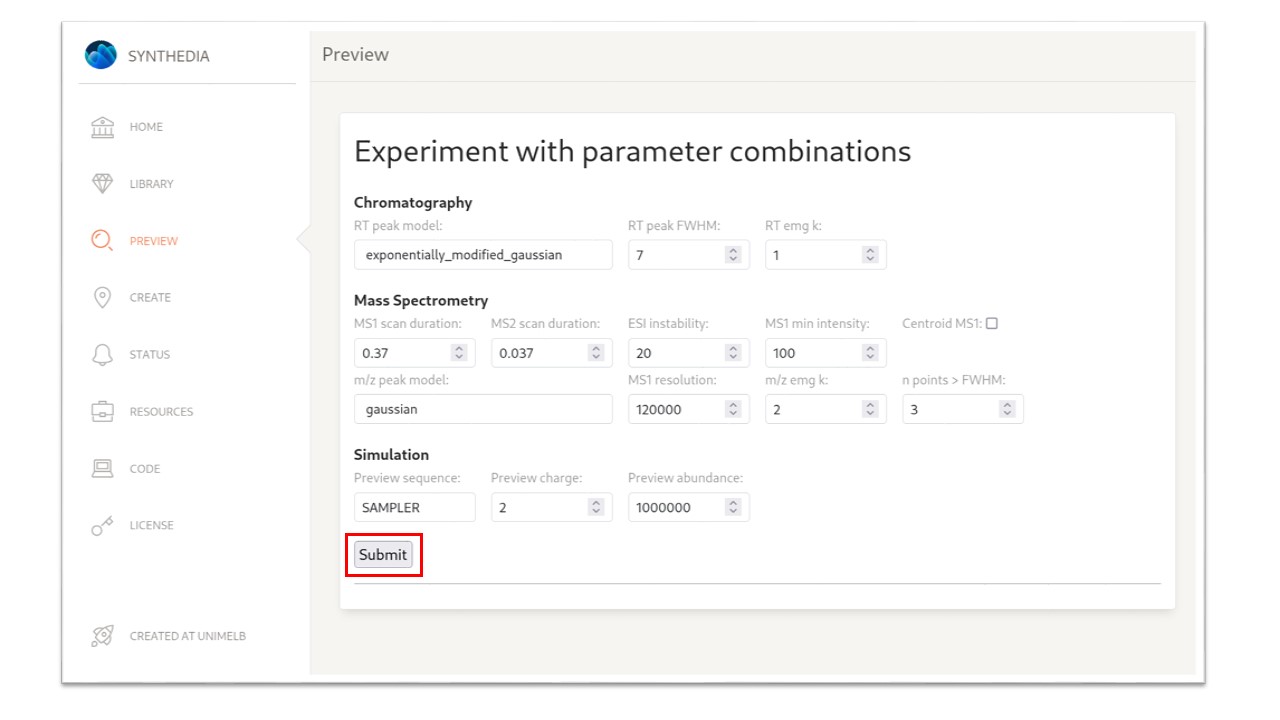


1. The page will be updated with images summarising the simulation of the single precursor ion. Here, we can see TIC traces for both MS1 and MS2 levels, an expansion of the monoisotopic MS1 precursor, a 2D heatmap of the MS1 precursor, and a graphic summarising the acquisition schema.


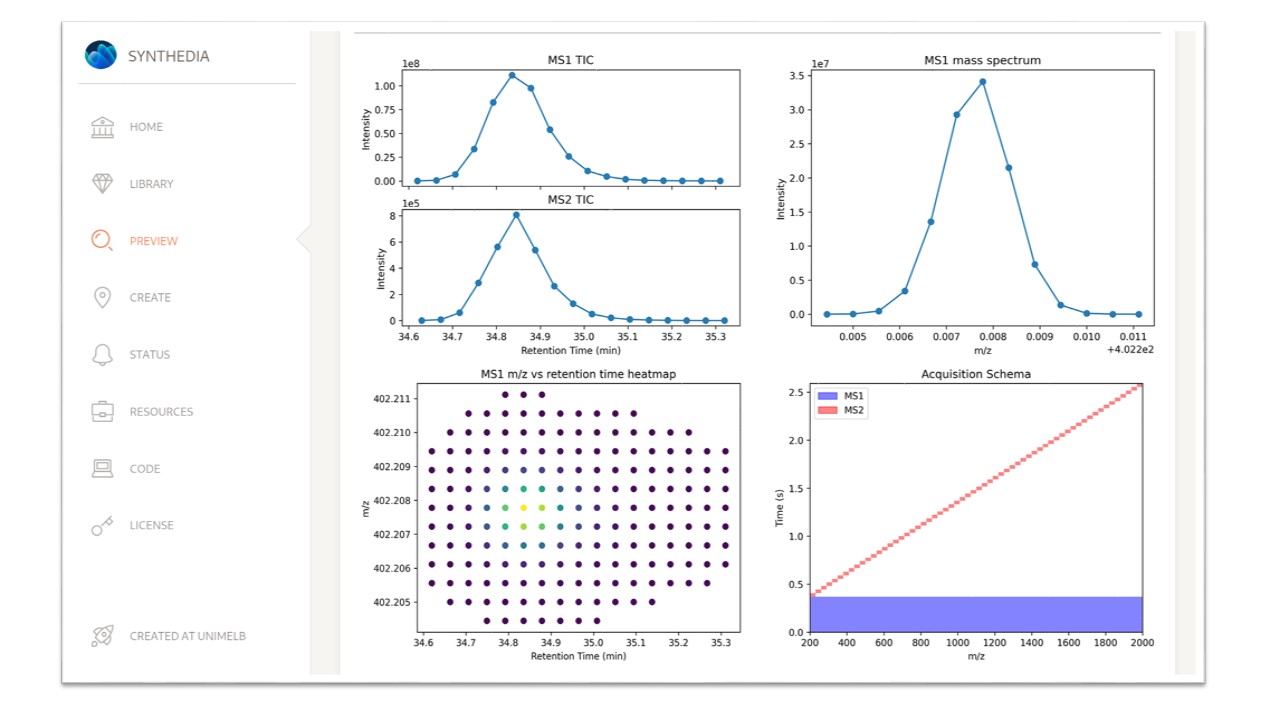

Supplement: vbac096_Supplementary_Data [file vbac096_supplementary_data.docx]
